# Development and validation a model for predicting overall survival of bladder cancer with lung metastasis: a population-based study

**DOI:** 10.1186/s40001-023-01261-w

**Published:** 2023-08-10

**Authors:** Liang Liu, Fu-zhen Sun, Pan-ying Zhang, Yu Xiao, Hai-xin Ni

**Affiliations:** 1 Department of Urology, Baoding No.1 Central Hospital, No.320 Changcheng North Street, Lianchi District, Baoding, 071000 Hebei China; 2 Prostate & Andrology Key Laboratory of Baoding, Baoding, China; 3 Department of Surgery and Urology, Hebei General Hospital, Shijiazhuang, China; 4 Psychosomatic Medical Center, The Fourth People’s Hospital of Chengdu, Chengdu, China; 5 Psychosomatic Medical Center, The Clinical Hospital of Chengdu Brain Science Institute, MOE Key Lab for Neuroinformation, University of Electronic Science and Technology of China, Chengdu, China

**Keywords:** Bladder cancer, Lung metastases, Nomogram, Overall survival

## Abstract

**Background:**

Although the number of patients with bladder cancer and lung metastasis is increasing there is no accurate model for predicting survival in these patients.

**Methods:**

Patients enrolled in the Surveillance, Epidemiology, and End Results database between 2010 and 2015 were selected for the study. Univariate and multivariate Cox regression were used to determine independent prognostic factors, followed by development of a nomogram based on the multivariate Cox regression models. The consistency index, receiver operating characteristic curve, and calibration curve were used to validate the prognostic nomogram.

**Results:**

506 eligible bladder cancer patients with lung metastasis were enrolled in the study and then divided randomly into training and validation sets (n = 356 vs. n = 150). Multivariate Cox regression analysis indicated that age at diagnosis, primary site, histological type, surgery of the primary site, chemotherapy, bone metastasis, and liver metastasis were prognostic factors for overall survival (OS) in patients with lung metastasis in the training set. The C-index of the nomogram OS was 0.699 and 0.747 in the training and validation sets, respectively. ROC curve estimation of the nomogram in the training and validation sets showed acceptable accuracy for classifying 1-year survival, with an area under the curve (AUC) of 0.766 and 0.717, respectively. More importantly, the calibration plot showed the nomogram had favorable predictive accuracy in both the training and validation sets.

**Conclusions:**

The prognostic nomogram created in our study provides an individualized diagnosis, remedy, and risk evaluation for survival in patients with bladder cancer and lung metastasis. The nomogram would therefore enable clinicians to make more precise treatment decisions for patients with bladder cancer and lung metastasis.

## Introduction

Cancer is not only the leading cause of death but is also the most important barrier to increased life expectancy in the world. Bladder cancer (BCa) is the tenth most common form of cancer globally, with an estimated 573,278 new BCa patients occurring in 185 countries during 2020, with 212,536 of these cases dying as a result of the tumor [1]. BCa included several subtypes such as urothelial carcinoma, squamous cell carcinoma, and adenocarcinoma. Among these, urinary bladder urothelial carcinoma was the major common histological subtype. Untreated metastatic bladder carcinoma has a poor prognosis, with the median survival time rarely exceeding 3 to 6 months [2].

Bladder carcinoma metastases include several patterns such as lymph node involvement (25.4%) and distant organ metastasis including bone (24.7%), brain (3.1%), liver (18.1%), and lung (19.4%). However, distant organ metastasis in BCa is considered a unique situation and although occurring rarely has an important significance as it is associated with significantly shortened survival [3–7]. Unfortunately, approximately 10–15% of BCa patients are diagnosed with distant metastases at the time of the initial diagnosis, with up to 30% of patients with high-grade BCa eventually developing metastases that lead to a poor prognosis [8]. BCa with distant metastases can be treated with immunotherapy and systemic chemotherapy, resulting in 5-year survival rates of 36% for regional metastasis and 5% with distant metastasis [9]. The overall survival (OS) rate of BCa patients with metastases remains quite low despite multiple therapeutic modalities. For this reason, it is essential to construct prognostic models for OS of BCa patients with lung metastasis, as identifying patients with evaluated poor survival outcomes may guide enhanced therapeutics and improve prognosis [10].

According to a previous analysis of the Surveillance, Epidemiology, and End Results (SEER) database, up to 38.9% (724/1862) of BCa patients with distant metastasis had lung metastases [11]. Considering the rarity of lung metastases at presentation, there are currently no randomized clinical trial that have investigated the outcomes of this group.

However, we are aware of a few studies on metastatic BCa that have focused on the prognostic significance of lung metastasis from BCa detected at de novo diagnosis. Prognostic nomograms are currently used widely in oncologic medicine as prognostic devices. Because the knowledge of the prognosis of lung metastasis is essential for pretherapeutic assessment the aim of the current study was to describe the frequency of occurrence based on the SEER database. Another aim of the study was to construct a nomogram to predict OS in de novo diagnosed patients with BCa and lung metastasis. The study may also help to choose suitable management strategies by increasing the understanding of prognosis in newly diagnosed patients with BCa and lung metastasis.

## Materials and methods

### Database and patient selection

All patient data in the study were selected from the National Cancer Institute-funded SEER database (http://seer.cancer.gov/seerstat), which includes approximately 28% of the population of the USA. The database includes clinicopathologic and demographic information and survival outcomes, such as age, sex, race, year of diagnosis, marital status at diagnosis, primary site, histological type, tumor grade, tumor-node metastasis (TNM) stage based on the American Joint Commission on Cancer (AJCC) 7th edition, treatment methods of the primary site, cause of death, and survival time [12]. Site‐specific metastasis data in the SEER database only includes the lung, brain, bone, and liver at diagnosis since 2010. The variable (C67.0–C67.9, positive histology, one primary, or 1st of 2 or more primaries) was used in the SEER*Stat software (version 8.3.9) to identify 76,686 BCa patients enrolled in the database between January 1, 2010 to December 31, 2015.

**Fig. 1:**
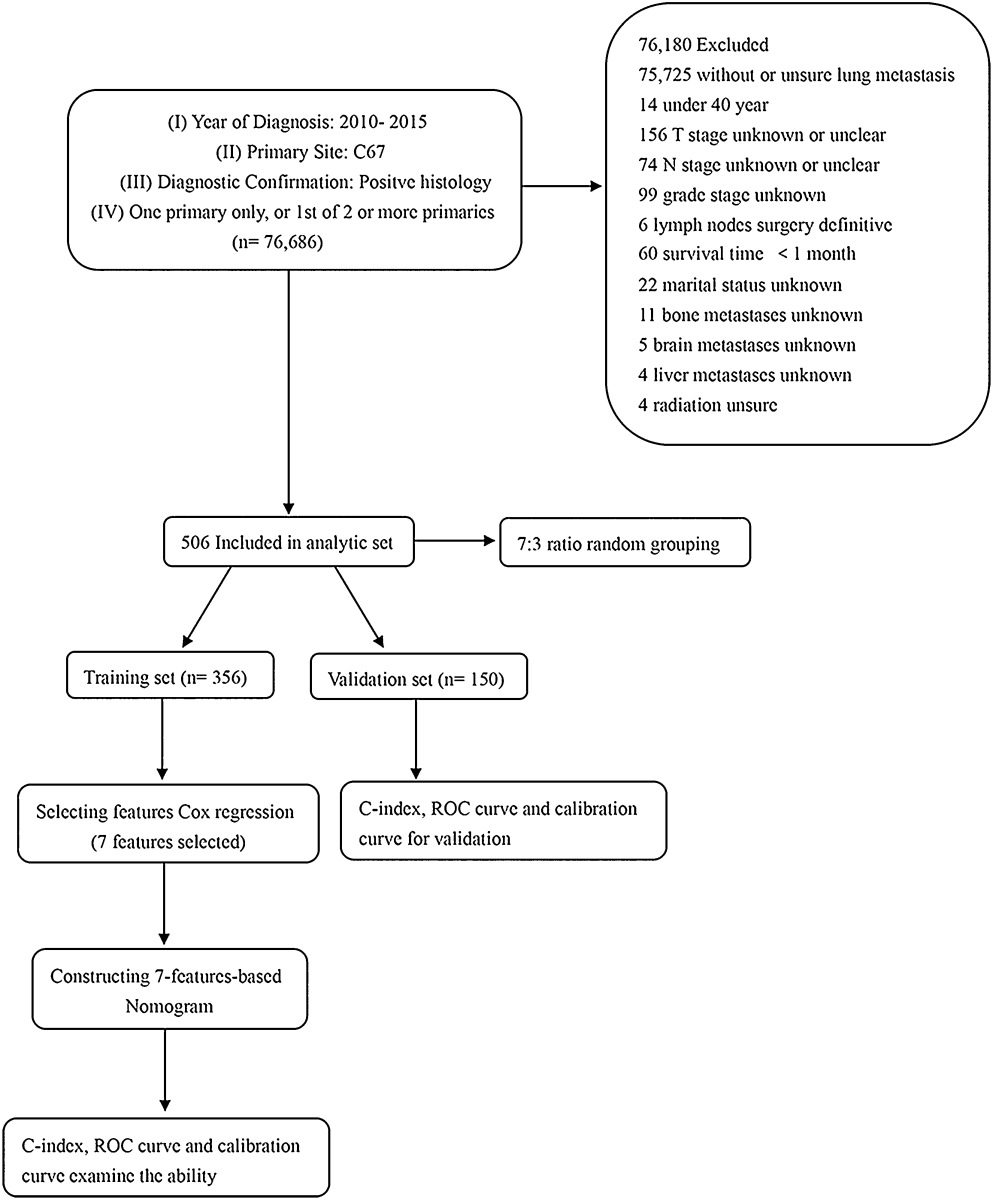
Schematic flowchart of the inclusion and exclusion criteria for this study

As shown in Fig. 1, the exclusion criteria for the study were as follows: (I) without or unsure of lung metastasis (n = 75,725); (II) patients aged < 40 years (n = 14); (III) T stage unknown or unclear (n = 156), N stage unknown or unclear (n = 74), grade stage unknown (n = 99); (IV) lymph nodes surgery definitive (n = 6); (V) survival time < 1 month (n = 60); (VI) marital status unknown (n = 22); and (VII) bone metastases unknown (n = 11), brain metastases unknown (n = 5), liver metastases unknown (n = 4); and (VIII) radiation unsure (n = 4).

Due to BCa occurring late in life, with a mean age at diagnosis of approximately 67 years, patients younger than 40 years were excluded for the number of reasons. Because the study was a retrospective analysis, potential selection bias and confounding bias were inevitable. Finally, 506 eligible BCa patients with lung metastasis were enrolled in the study. The study was performed in compliance with the Declaration of Helsinki.

### Study variables

The variables included the participants’ sex, year of diagnosis, ethnicity, marital status at diagnosis, primary site, histology type, grade stage, TNM stage, surgery of the primary site, surgery of lymph nodes, surgery of other sites, radiotherapy, chemotherapy, distant metastatic site, survival months, and vital status. The primary end point was OS according to the database. OS was defined as the time from diagnosis to death from any cause.

For convenient analysis we processed some variables in the SEER database using continuous variables for radiation, chemotherapy, bone metastasis, liver metastasis, brain metastasis, and surgery of another site, classified as either yes or no. Age was transformed into categorical variables: < 50, 50–59, 60–69, 70–79, or ≥ 80 years. Ethnicity included black, white, and others which included American, Native, Asian, Alaska, and Pacific Island people. We defined marital status as unmarried, married, divorced, separated, or widowed. The histology of the tumors in the study patients included transitional cell carcinoma, squamous cell carcinoma, adenocarcinoma, and other types. Precise information on the TMN system was recorded based on the seven editions of the AJCC stages.

### Statistical analysis

All cases were included in the study set, which was then divided into training and validation sets at a ratio of 7:3. Categorical data were expressed as numbers and percentage in the three datasets, with the chi-square test used for intergroup comparisons. Continuous variables were expressed as mean ± standard deviation (SD), with Student’s t-test used to compare the baseline characteristics of the patients in the two groups. Categorical variables were expressed as frequencies and proportions and comparisons carried out using the Chi-square test.

A nomogram incorporating selected variables was constructed from the multiple Cox model, using a critical *P*-value of 0.05. To ascertain the risk factors associated with the OS of BCa patients with lung metastasis at diagnosis, we determined the hazard rations (HR) and 95%CI for the training set using univariate and multivariable Cox proportional analyses. The survival curves were plotted using the Kaplan–Meier curve and then compared using the log-rank test.

Subsequently, based on the results of the multivariate Cox regression model, we used the rms-R package to construct a prognostic nomogram to predict OS probability in BCa patients with lung metastasis. In addition, the consistency index (C-index), receiver operating characteristic (ROC) curve, and calibration curve were used to estimate the predictive performance of the nomogram, and also to calibrate the prediction capacity of the nomogram for 1- and 3-year OS. The C-index and an area under the curve (AUC) of > 0.65 in the receiver operating characteristic graph were considered to indicate acceptable classification. The calibration plot was used to assess the predictive accuracy by comparison with the actual survival rate of the nomogram in the training set. Similarly, the C-index, ROC curve, and calibration curve were used to analyze the validation set. The statistical analyses were carried out using the SPSS software program (version 25.0) and R software (www.rproject.org). *P* values < 0.05 indicated statistical significance.

## Results

### Demographic and clinicopathologic characteristics

A total of 506 (training cohort, 356 patients; validation cohort, 150 patients) BCa patients with lung metastasis enrolled in the database between January 1, 2010 and December 31, 2015, were eligible for selection in the study cohort. The clinicopathological features and demographic characteristics of these 506 patients are described in Table 1. The differences in clinical data between the training and validation set were not statistically significant (*P* > 0.05). The majority of patients in both cohorts were aged 60–80 years, married, and white. There were 154 (30.4%) females and 352 (69.6%) males in the full study cohort, with the most common tumor locations being bladder, NOS (209, 41.3%), followed by overlapping lesions of the bladder (113, 22.3%) and lateral wall of the bladder (72, 14.2%). The most common tumors were transitional cell carcinoma (448, 88.5%), T2 stage (246, 48.6%), and N0 stage (314, 62.1%). There were 123 (24.3%) bone metastasis, 15 (3.0%) brain metastasis, and 96 (19.0%) liver metastasis in the full study cohort. The median survival time was 6 months (range, 1–98 months).

**Table 1:** Baseline characteristics of patients in the training and validation sets

Characteristics	Total (n = 506)	Training set (n = 356)	Validation set (n = 150)	*P* value
Age (year), n (%)				0.490
< 50	26 (5.1)	18 (5.1)	8 (5.3)	
50–59	83 (16.4)	56 (15.7)	27 (18.0)	
60–69	126 (24.9)	87 (24.4)	39 (26.0)	
70–79	157 (31.0)	119 (33.4)	38 (25.3)	
≥ 80	114 (22.5)	76 (21.3)	38 (25.3)	
Sex, n (%)				0.941
Female	154 (30.4)	108 (30.3)	46 (30.7)	
Male	352 (69.6)	248 (69.7)	104 (69.3)	
Marital status, n (%)				0.283
Unmarried	96 (19.0)	62 (17.4)	34 (22.7)	
Married	260 (51.4)	186 (52.2)	74 (49.3)	
Divorced	67 (13.2)	49 (13.8)	18 (12.0)	
Separated	7 (1.4)	7 (2.0)	0 (0.0)	
Widowed	76 (15.0)	52 (14.6)	24 (16.0)	
Race, n (%)				0.859
Black	58 (11.5)	39 (11.0)	19 (12.7)	
White	424 (83.8)	300 (84.3)	124 (82.7)	
Other	24 (4.7)	17 (4.8)	7 (4.7)	
Primary site, n (%)				0.371
Trigone of bladder	23 (4.5)	19 (5.3)	4 (2.7)	
Dome of bladder	18 (3.6)	12 (3.4)	6 (4.0)	
Lateral wall of bladder	72 (14.2)	47 (13.2)	25 (16.7)	
Anterior wall of bladder	8 (1.6)	7 (2.0)	1 (0.7)	
Posterior wall of bladder	35 (6.9)	29 (8.1)	6 (4.0)	
Bladder neck	17 (3.4)	14 (3.9)	3 (2.0)	
Ureteric orifice	9 (1.8)	7 (2.0)	2 (1.3)	
Urachus	2 (0.4)	1 (0.3)	1 (0.7)	
Overlapping lesion of bladder	113 (22.3)	81 (22.8)	32 (21.3)	
Bladder, NOS	209 (41.3)	139 (39.0)	70 (46.7)	
Grade stage, n (%)				0.322
Well differentiated	7 (1.4)	4 (1.1)	3 (2.0)	
Moderately differentiated	24 (4.7)	19 (5.3)	5 (3.3)	
Poorly differentiated	130 (25.7)	85 (23.9)	45 (30.0)	
Undifferentiated	345 (68.2)	248 (69.7)	97 (64.7)	
Histologic type, n (%)				0.484
Transitional	448 (88.5)	319 (89.6)	129 (86.0)	
Squamous	24 (4.7)	16 (4.5)	8 (5.3)	
Adenocarcinoma	11 (2.2)	8 (2.2)	3 (2.0)	
Other	23 (4.5)	13 (3.7)	10 (6.7)	
AJCC T, n (%)				0.681
T1	103 (20.4)	72 (20.2)	31 (20.7)	
T2	246 (48.6)	177 (49.7)	69 (46.0)	
T3	55 (10.9)	35 (9.8)	20 (13.3)	
T4	102 (20.2)	72 (20.2)	30 (20.0)	
AJCC N, n (%)				0.795
N0	314 (62.1)	222 (62.4)	92 (61.3)	
N1	65 (12.8)	48 (13.5)	17 (11.3)	
N2	99 (19.6)	68 (19.1)	31 (20.7)	
N3	28 (5.5)	18 (5.1)	10 (6.7)	
Surgery of primary site, n (%)				0.667
None	39 (7.7)	24 (6.7)	15 (10.0)	
TURB	349 (69.0)	247 (69.4)	102 (68.0)	
Partial cystectomy	3 (0.6)	2 (0.6)	1 (0.7)	
Radical cystectomy	26 (5.1)	18 (5.1)	8 (5.3)	
Pelvic exenteration	26 (5.1)	17 (4.8)	9 (6.0)	
Other	63 (12.5)	48 (13.5)	15 (10.0)	
Surgery of lymph nodes, n (%)				0.177
None	456 (90.1)	323 (90.7)	133 (88.7)	
1 to 3 removed	5 (1.0)	5 (1.4)	0 (0.0)	
4 or more removed	45 (8.9)	28 (7.9)	17 (11.3)	
Radiation, n (%)				0.100
No	402 (79.4)	276 (77.5)	126 (84.0)	
Yes	104 (20.6)	80 (22.5)	24 (16.0)	
Chemotherapy, n (%)				0.908
No	228 (45.1)	161 (45.2)	67 (44.7)	
Yes	278 (54.9)	195 (54.8)	83 (55.3)	
Bone metastasis, n (%)				0.903
No	383 (75.7)	270 (75.8)	113 (75.3)	
Yes	123 (24.3)	86 (24.2)	37 (24.7)	
Brain metastasis, n (%)				0.587
No	491 (97.0)	344 (96.6)	147 (98.0)	
Yes	15 (3.0)	12 (3.4)	3 (2.0)	
Liver metastasis, n (%)				0.391
No	410 (81.0)	285 (80.1)	125 (83.3)	
Yes	96 (19.0)	71 (19.9)	25 (16.7)	
Surgery of other site, n (%)				0.975
No	472 (93.3)	322 (93.3)	140 (93.3)	
Yes	34 (6.7)	24 (6.7)	10 (6.7)	

### Identification of prognostic factors of OS

**Fig. 2:**
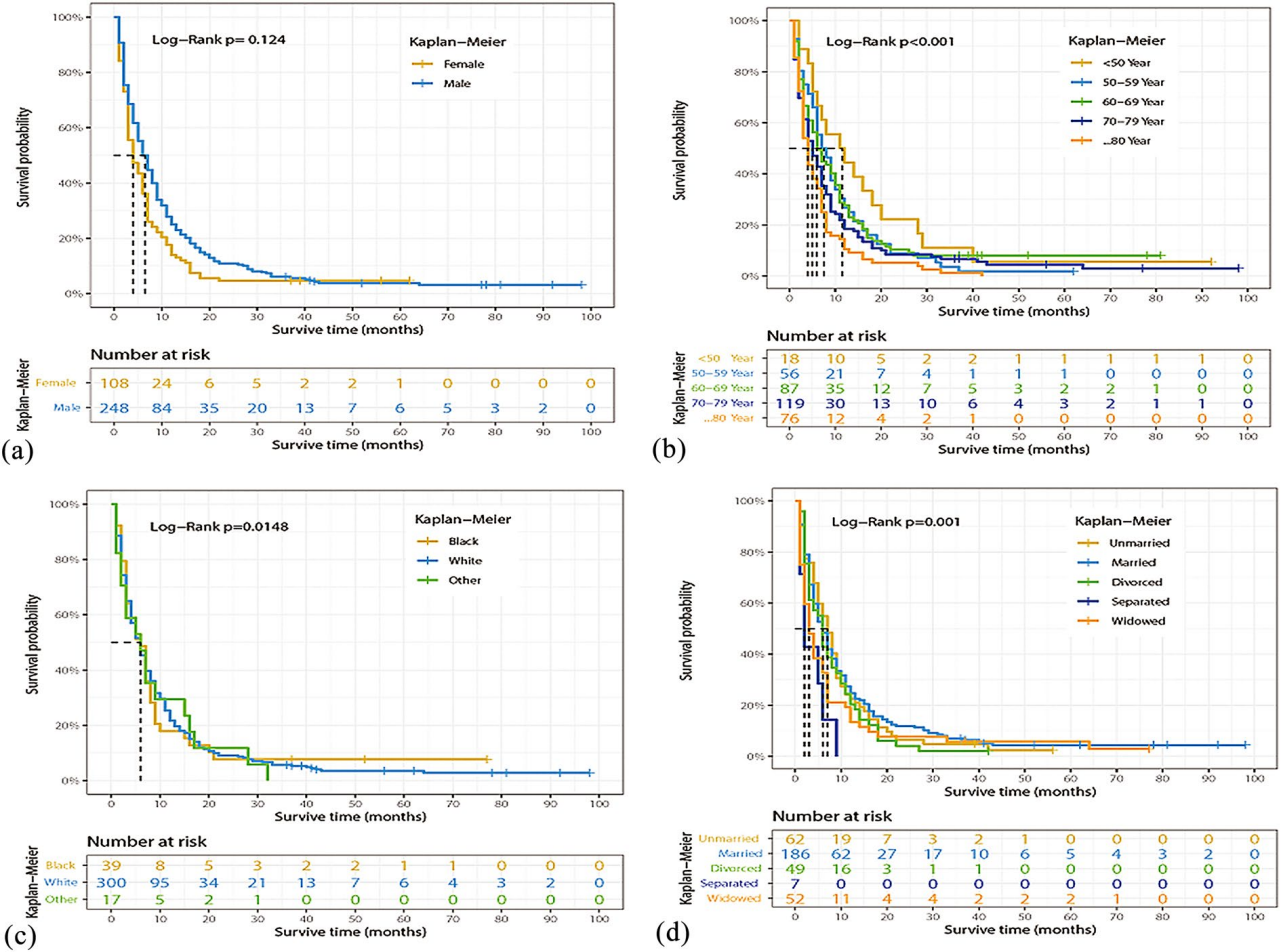
Kaplan–Meier estimated overall survival in BCa patients with lung metastasis stratified by sex (**a**), age (**b**), race (**c**), marital status (**d**)

**Fig. 3:**
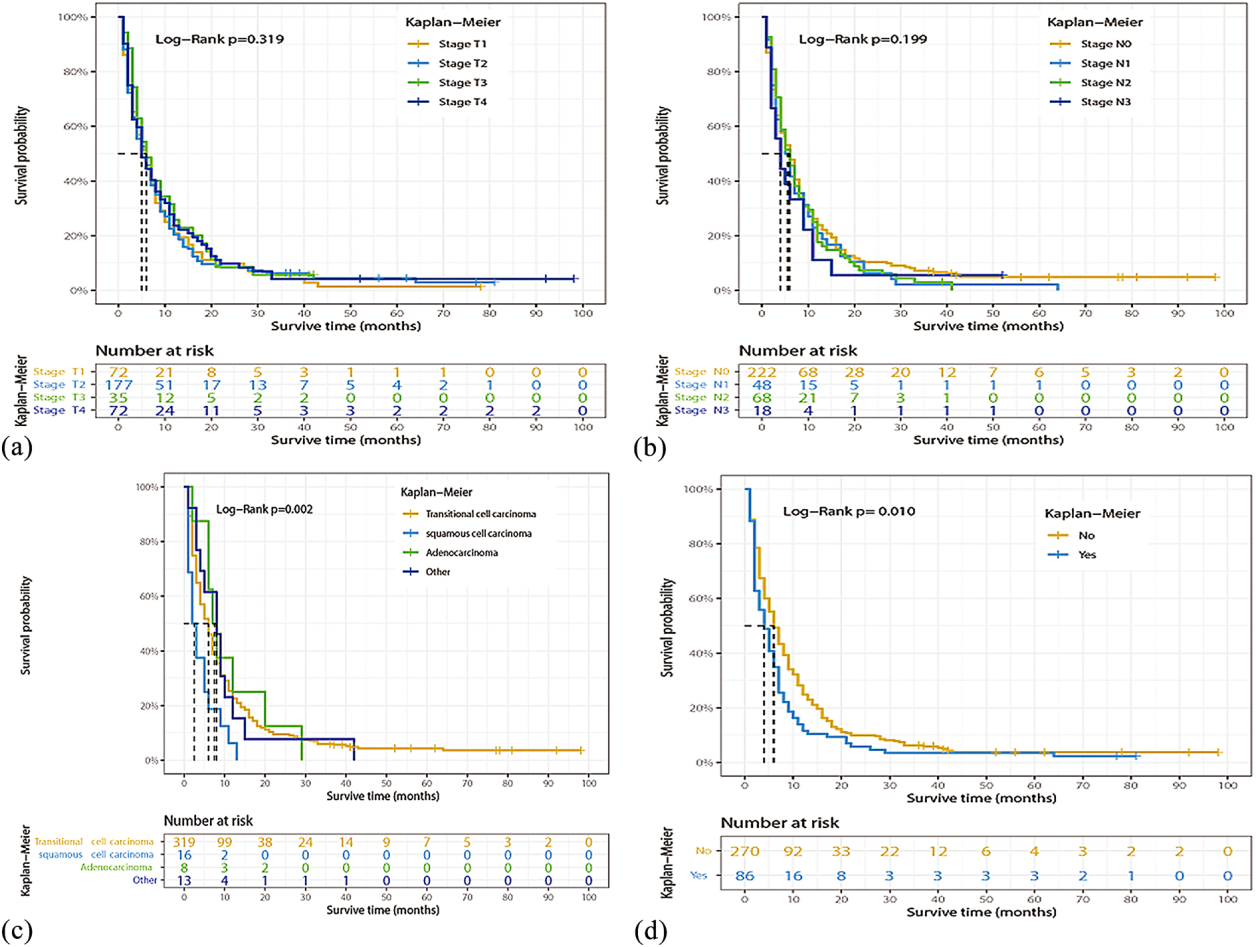
Kaplan–Meier estimated overall survival in BCa patients with lung metastasis stratified by stage T (**a**), stage N (**b**), histologic type (**c**), bone metastasis (**d**)

**Fig. 4:**
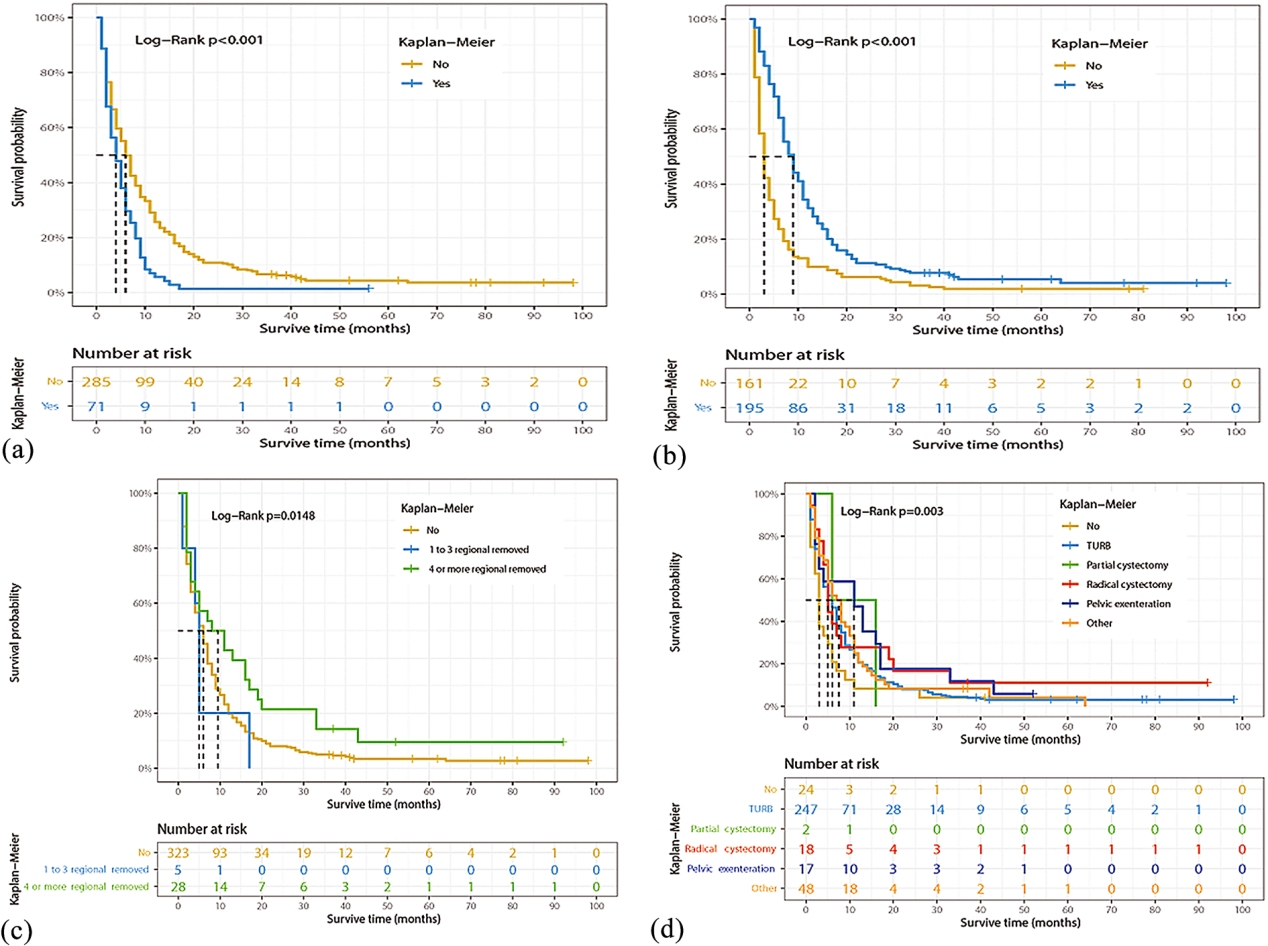
Kaplan–Meier estimated overall survival in BCa patients with lung metastasis stratified by liver metastasis (**a**), chemotherapy (**b**), lymph node surgery (**c**), surgery of primary site (**d**)

Univariate and multivariate Cox regression models were performed on all factors in the training set, with the exception of the year of diagnosis. Univariate Cox regression analysis showed that age at diagnosis, sex, marital status, histological type, surgery of the primary site, surgery of lymph nodes, chemotherapy, bone metastasis, and liver metastasis were factors related to OS in BCa patients with lung metastasis. Multivariate Cox regression model results also showed that age at diagnosis, primary site, histological type, surgery of the primary site, chemotherapy, bone metastasis, and liver metastasis were prognostic factors for OS in these patients (Table 2). For example, higher age (70–79 years: HR = 1.936, 95%CI 1.074–3.490, *P* < 0.05; ≥ 80 years: HR = 2.361, 95%CI 1.244–4.481, *P* < 0.05), tumor located in the dome of bladder (HR = 2.352, 95%CI 1.028–5.381, *P* < 0.05), squamous cell carcinoma (HR = 6.458, 95%CI 3.121–13.364, *P* < 0.001), and combined with bone (HR = 1.437, 95%CI 1.096–1.885, *P* < 0.05) or liver (HR = 1.624, 95%CI 1.187–2.223, *P* < 0.05) metastasis were associated with a worse OS. In contrast, surgery of the primary site [transurethral resection of the bladder (TURB): HR = 0.548, 95%CI 0.337–0.890, *P* < 0.05; other type: HR = 0.423, 95%CI 0.238–0.752, *P* < 0.05], or chemotherapy (HR = 0.465, 95%CI 0.359–0.604, *P* < 0.001) were associated with a favorable OS. Figures 2, 3 and 4 show the Kaplan–Meier curves for these relevant variables.

**Table 2:** Univariate and multivariate Cox regression analysis of BCa patients with lung metastasis based on clinicopathological characteristics data in the training cohort

Characteristics	Univariate analysis HR (95% CI)	*P* value	Multivariate analysis HR (95% CI)	*P* value
Age (year), n (%)				
< 50	Ref		Ref	
50–59	1.409 (0.817–2.429)	0.217	1.425 (0.761–2.669)	0.268
60–69	1.365 (0.808–2.304)	0.245	1.603 (0.887–2.898)	0.118
70–79	1.684 (1.011–2.805)	0.045*	1.936 (1.074–3.490)	0.028*
≥ 80	2.267 (1.335–3.847)	0.002*	2.361 (1.244–4.481)	0.009*
Sex, n (%)				
Female	Ref		Ref	
Male	0.740 (0.586–0.934)	0.011*	0.853 (0.643–1.132)	0.270
Marital status, n (%)				
Unmarried	Ref		Ref	
Married	0.961 (0.717–1.288)	0.790	1.156 (0.830–1.609)	0.392
Divorced	1.141 (0.780–1.667)	0.498	1.223 (0.800–1.871)	0.354
Separated	2.497 (1.137–5.483)	0.023*	1.532 (0.632–3.712)	0.345
Widowed	1.380 (0.947–2.010)	0.094	1.250 (0.808–1.933)	0.317
Race, n (%)				
Black	Ref		Ref	
White	1.008 (0.713–1.426)	0.963	0.931 (0.625–1.388)	0.727
Other	1.059 (0.594–1.886)	0.847	1.428 (0.759–2.686)	0.269
Primary site, n (%)				
Trigone of bladder	Ref		Ref	
Dome of bladder	1.214 (0.589–2.501)	0.600	2.352 (1.028–5.381)	0.043*
Lateral wall of bladder	0.943 (0.549–1.619)	0.832	1.433 (0.784–2.619)	0.243
Anterior wall of bladder	0.891 (0.355–2.237)	0.806	2.375 (0.888–6.349)	0.085
Posterior wall of bladder	1.348 (0.748–2.428)	0.320	1.651 (0.839–3.252)	0.147
Bladder neck	0.970 (0.479–1.966)	0.933	1.263 (0.586–2.719)	0.551
Ureteric orifice	1.070 (0.449–2.547)	0.879	1.788 (0.694–4.610)	0.229
Urachus	0.505 (0.068–3.778)	0.506	1.925 (0.188–19.74)	0.581
Overlapping lesion of bladder	1.211 (0.735–1.997)	0.452	1.749 (0.987–3.097)	0.055
Bladder, NOS	1.144 (0.707–1.851)	0.584	1.728 (0.998–2.991)	0.051
Grade stage, n (%)				
Well differentiated	Ref		Ref	
Moderately differentiated	0.556 (0.187–1.655)	0.291	1.459 (0.423–5.029)	0.550
Poorly differentiated	0.662 (0.242–1.811)	0.422	3.404 (0.958–12.100)	0.058
Undifferentiated	0.702 (0.261–1.888)	0.483	3.366 (0.956–11.854)	0.059
Histologic type, n (%)				
Transitional	Ref		Ref	
Squamous	2.165 (1.304–3.593)	0.003*	6.458 (3.121–13.364)	< 0.001^#^
Adenocarcinoma	0.833 (0.413–1.682)	0.611	1.375 (0.571–3.312)	0.478
Other	0.972 (0.558–1.694)	0.920	0.844 (0.455–1.567)	0.592
AJCC T, n (%)				
T1	Ref		Ref	
T2	0.982 (0.744–1.296)	0.899	1.031 (0.759–1.401)	0.845
T3	0.839 (0.555–1.268)	0.405	0.866 (0.528–1.423)	0.571
T4	0.910 (0.654–1.268)	0.579	0.810 (0.540–1.217)	0.311
AJCC N, n (%)				
N0	Ref		Ref	
N1	1.135 (0.829–1.554)	0.429	1.210 (0.837–1.749)	0.312
N2	1.095 (0.831–1.443)	0.518	1.248 (0.900–1.731)	0.185
N3	1.288 (0.785–2.113)	0.317	1.567 (0.918–2.674)	0.100
Surgery of primary site, n (%)				
None	Ref		Ref	
TURB	0.646 (0.421–0.992)	0.046*	0.548 (0.337–0.890)	0.015*
Partial cystectomy	0.493 (0.116–2.092)	0.337	0.503 (0.096–2.636)	0.416
Radical cystectomy	0.494 (0.260–0.936)	0.031*	0.431 (0.115–1.624)	0.214
Pelvic exenteration	0.438 (0.231–0.831)	0.012*	0.457 (0.122–1.704)	0.243
Other	0.581 (0.352–0.960)	0.034*	0.423 (0.238–0.752)	0.003*
Surgery of lymph nodes, n (%)				
None	Ref		Ref	
1 to 3 removed	1.298 (0.536–3.143)	0.564	1.359 (0.290–6.373)	0.698
4 or more removed	0.621 (0.412–0.935)	0.023*	0.791 (0.240–2.608)	0.700
Radiation, n (%)				
No	Ref		Ref	
Yes	1.247 (0.967–1.608)	0.089	1.19 (0.899–1.589)	0.221
Chemotherapy, n (%)				
No	Ref		Ref	
Yes	0.477 (0.385–0.592)	< 0.001^#^	0.465 (0.359–0.604)	< 0.001^#^
Bone metastasis, n (%)				
No	Ref		Ref	
Yes	1.391 (1.086–1.782)	0.009*	1.437 (1.096–1.885)	0.009*
Brain metastasis, n (%)				
No	Ref		Ref	
Yes	1.512 (0.848–2.694)	0.161	1.608 (0.831–3.115)	0.159
Liver metastasis, n (%)				
No	Ref		Ref	
Yes	1.723 (1.319–2.26)	< 0.001^#^	1.624 (1.187–2.223)	0.002*
Surgery of other site, n (%)				
No	Ref		Ref	
Yes	0.780 (0.524–1.222)	0.301	0.997 (0.594–1.673)	0.990

^*^P < 0.05; ^#^*P* < 0.001

### Development of a prognostic nomogram

**Fig. 5:**
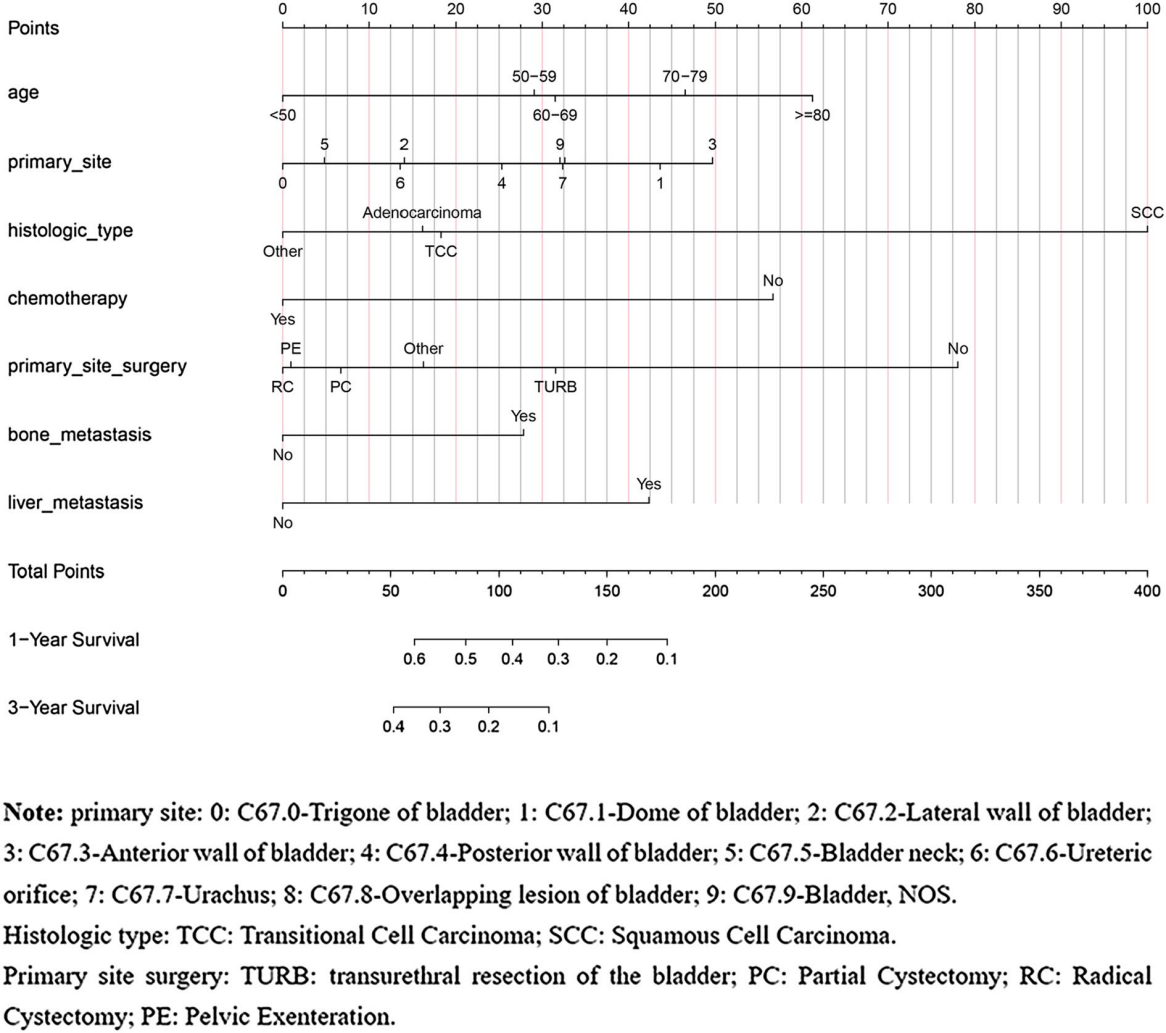
Prognostic nomogram predicting 1- and 3-year overall survival rate in patients with lung metastatic BCa

The variables with a *P* value < 0.05 in the multivariate Cox regression models were included in the prognostic nomogram. We constructed the nomogram using the prognostic factors identified in the multivariate Cox regression model of the training set. The TNM stage was an important prognostic factor for tumor patients, although it did not show statistically significant power to predict outcomes in BCa patients with lung metastasis. The results of the multivariate Cox regression models showed that T stage (*P* > 0.05) and N stage (*P* > 0.05) were not prognostic factors for OS in BCa patients with lung metastasis and therefore we did not integrate TNM stage into the nomogram. The nomogram based on the prognostic factors is shown in Fig. 5. Using this nomogram individual survival at 1- and 3-year could be predicted by the available clinical information.

Each subgroup variable could get a corresponding score in the nomogram. The scores ranged from 0 to 100 for each variable depending on its contribution, and produced total scores of the subscales that were then transformed to predict the related OS (Table 3). Using the nomogram, a vertical line is drawn up to the top row of the points and points assigned for each variable. The sum of the scores is located on the total points axis, and a downward line drawn on the survival axis to determine the likelihood of survival for 1- or 3-years.

**Table 3:** The approximate risk point of each variable and computational formula of OS

Clinical variables	Values	Risk points
Age (year)	< 50	0
	50–59	29
	60–69	32
	70–79	46
	≥ 80	62
Primary site	Trigone of bladder	0
	Dome of bladder	43
	Lateral wall of bladder	14
	Anterior wall of bladder	50
	Posterior wall of bladder	26
	Bladder neck	5
	Ureteric orifice	13
	Urachus	33
	Overlapping lesion of bladder	33
	Bladder, NOS	32
Histologic type	Transitional	18
	Squamous	100
	Adenocarcinoma	16
	Other	0
Surgery of primary site	None	78
	TURB	32
	Partial cystectomy	7
	Radical cystectomy	0
	Pelvic exenteration	1
	Other	16
Chemotherapy	No	57
	Yes	0
Bone metastasis	No	0
	Yes	28
Liver metastasis	No	0
	Yes	42

### Validation and calibration of the nomograms

**Fig. 6:**
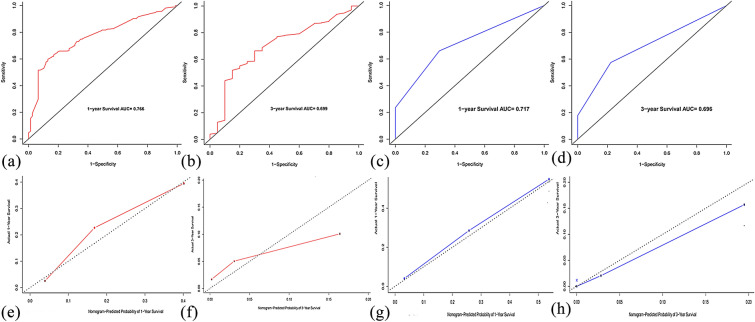
The ROC curve for predicting patient survival at 1 years (**a**, **c**) and 3 years (**b**, **d**) in the training and validation set. The calibration curve for predicting patient survival at 1 years (**e**, **g**) and 3 years (**f**, **h**) in the training and validation set

The C-index and ROC curves were compared to determine, whether the survival months predicted by the nomograms were in accordance with the actual survival times. The C-index of the nomogram OS was 0.699 and 0.747 in the training and validation sets, respectively. The ROC curve estimation of the nomogram in both the training and validation sets also showed acceptable accuracy, with a 1-year AUC of 0.766 and 0.717, respectively (Fig. 6a, c). In addition, the 3-year AUCs were 0.699 and 0.696, respectively (Fig. 6b, d). These results indicated that the model we constructed was relatively accurate.

More importantly, we calibrated the 1- and 3-year OS nomogram in both the training and validation sets. The calibration plots showed that the nomogram had a favorable predictive accuracy in both the training set (Fig. 6e, f) and validation set (Fig. 6g, h). This result indicated good agreement between the nomogram predictions and the observed results in the training and validation sets.

## Discussion

To our knowledge, a considerable number of BCa patients have metastases at distant organs at diagnosis, leading to a shorter OS. Of the metastatic BCa organs, bone is the most common, followed by the lung. Bianchi et al. [4] also showed that the bone metastasis rate was higher than the lung metastasis rate in patients with M1 stage bladder cancer. A previous study studied the independent prognostic factors of outcome in BCa patients with bone metastasis [13]. However, the independent predictive factors of outcome in lung metastasis BCa still remain unknown. The aim of the present research was therefore to determine the independent predictive factors in BCa patients with lung metastasis and develop a predictive nomogram to help predict outcome risks.

Univariate and multivariate analyses were carried out in the current study on a large number of BCa patients with lung metastasis. These analyses showed that age at diagnosis, primary site of the tumor, histology, surgery of the primary site, additional chemotherapy, bone metastasis, and liver metastasis were independent prognostic factors for OS in BCa patients with lung metastasis.

The nomogram we constructed enables more personalized risk prediction and is a well-studied intuitive statistical model based on the results of a multivariate analysis [14, 15]. The integration of multiple independent prognostic factors in the model could further improve its accuracy to appraise the survival probability of an individual patient [16]. To date, several nomograms have been developed for different tumor types and have shown powerful predictive ability that is more accurate than the traditional TNM systems [17]. More importantly, clinicians are able to intuitively evaluate the physical condition of patients and offer individual predictions using nomograms. However to our knowledge before the current study there was no prognostic nomogram model for BCa patients with lung metastasis. Therefore, it is of great prognostic significance for these patients to establish a reliable and efficacious prognostic nomogram and to offer individualized therapies.

The present study investigated and validated a new prognostic tool based on the results of multivariate Cox regression models that included the age at diagnosis, primary site of the tumor, histology, bone metastasis, liver metastasis, chemotherapy, and surgery of the primary site. This tool enhanced prediction of OS in BCa patients with lung metastases. The results of the study demonstrated that the prognostic tool could be used to divide the patients into two groups (low-risk and high-risk) with wide variations in OS. Using a median cut-off value, the patients were divided into either high-risk or low-risk groups. We also assessed the accuracy of the 1- and 3-year OS in the prognostic nomograms, with the results indicating good consistency and reliability. In terms of its content, the prediction model is simple and easy to understand. First, in the nomogram, a vertical line is drawn from each clinicopathological parameter to the ‘points’ line, followed by addition of the score to determine the ‘total points’. A vertical line is then drawn from ‘total points’ to ‘1- or 3-years survival’. Based on the above analysis we are able to calculate the 1- or 3-year survival rates in lung metastatic BCa patients. For example, a 65-year-old male patient was diagnosed with a transitional cell carcinoma located in the lateral wall of bladder, without liver or bone metastases, and had undergone a TURB and chemotherapy. According to the nomogram, the ‘total points’ was 95 and the 1- and 3-years survival rates were approximately 45% and 20%, respectively.

In general, the prognostic factors identified in our study were associated strongly with the choice of therapeutic approach, as well as the metastatic site of the BCa patients. Previous research in BCa patients has demonstrated that a single metastatic site was capable of independently predicting better OS compared with multisite organ metastasis [17]. The results of our research were consistent with this previous study in that it showed better survival with lung metastases compared to a coalesced tumor or simultaneous bone metastasis or liver metastasis.

Treatment of BCa patients with lung metastases is not uniform. In the current study, we included five variables in the treatment of lung metastasis BCa, including surgery of the primary site, surgery of lymph nodes, surgery of other sites, radiation, and chemotherapy.

A previous study revealed that the primary site of surgery might contribute to long-term OS survival in lung metastatic BCa patients. Wang et al. [18] reported that the survival rates of metastatic BCa patients with adenocarcinoma and transitional cell (non)-papillary carcinoma could be improved by surgery of the primary site alone, while the survival rates of metastatic BCa patients with squamous cell carcinoma could be increased only by surgery combined with adjuvant chemotherapy. They also demonstrated that surgery of the primary site alone was an independent predictive factor of outcome, and that surgery could affect the OS of BCa patients with lung metastasis. We found that BCa patients with lung metastasis could also benefit from primary site surgery. Regarding surgery of lung metastasis, the conclusions remain limited due to a lack of consistent reports. However, there is evidence that a pulmonary metastasectomy, combined with chemotherapy may improve OS [19]. Although the efficacy of therapeutic approaches is susceptible to the underlying emergence of drug resistance, as shown in our nomogram, chemotherapy still has the greatest survival benefit for BCa patients with lung metastasis. This finding is consistent with the first-line treatment regimen in the guidelines of the European Urological Association [20]. Besides, in cancer, fibroblast growth factor receptors (FGFRs) have emerged as a novel therapeutic target [21]. As an oncogenic driver in bladder cancer, FGFR3 genomic alterations represent predictive biomarkers that predict the response to FGFR inhibitors. According to research, 50% of bladder cancers have somatic mutations of the FGFR3 gene [22, 23]. Bladder cancer is typically associated with FGFR3 gene rearrangements [24, 25]. While platinum-based chemotherapies have always been the main therapeutic approach for urothelial bladder carcinoma, the advent of antiFGFR-target therapy represents significant advances for bladder cancer patients with FGFR altered.

To our knowledge this is the first cohort study to investigate the risk factors for the prognosis of BCa with lung metastasis. Furthermore, the study is the largest cohort study to examine the prognostic significance of lung metastasis in BCa patients and to determine the effect of numerous treatment modalities on the prognosis of these patients. Nevertheless, we acknowledge that our study has several limitations. First, the retrospective research design of the study limited its conclusions and it was not possible to completely rule out confounding factors, such as smoking history etc. In addition, information on cancer recurrence was lacking, and patients who might have developed distant metastases later in their disease process were not taken into consideration.

## Conclusions

Age at diagnosis, primary site of the tumor, histology, surgery of the primary site, additional chemotherapy, bone metastasis, and liver metastasis are independent predictive factors of outcome in BCa patients with lung metastasis. Based on these prognostic factors, we constructed a prognostic nomogram, which could provide the best assessment of OS and indicate appropriate therapy in BCa patients with lung metastasis.

## Data Availability

The data analyzed in this study is available at https://seer.Cancer.gov/.

## Abbreviations

OS: Overall survival
AUC: Area under the curve
BCa: Bladder cancer
SEER: Surveillance, Epidemiology, and End Results
TNM: Tumor-node metastasis
AJCC: American Joint Commission on Cancer
HR: Hazard rations
C-index: Consistency index
ROC: Receiver operating characteristic
TURB: Transurethral resection of the bladder
FGFR: Fibroblast growth factor receptors
